# Knowledge, Attitude and Practices of Abattoir Workers in Kumasi Towards Ticks and Tick‐Borne Pathogens

**DOI:** 10.1002/puh2.70167

**Published:** 2025-11-14

**Authors:** Seth Offei Addo, Margaret Addo, Michael E. DeWitt, Christopher Nii Laryea Tawiah‐Mensah, Stacy Amoah, Patrick Kwasi Obuam, Nancy Martekai Unicorn, Emmanuella Tiwaa Kyeremateng, Genevieve Desewu, John Asiedu Larbi

**Affiliations:** ^1^ Parasitology Department Noguchi Memorial Institute For Medical Research University of Ghana Accra Ghana; ^2^ Department of Medical Microbiology, University of Ghana Medical School University of Ghana Accra Ghana; ^3^ Department of Internal Medicine Section on Infectious Diseases Wake Forest University School of Medicine Winston‐Salem North Carolina USA; ^4^ Center For the Study of Microbial Ecology and Emerging Diseases Wake Forest University School of Medicine Winston‐Salem North Carolina USA; ^5^ Department of Biology Wake Forest University Winston‐Salem North Carolina USA; ^6^ Department of Theoretical and Applied Biology College of Science KNUST Kumasi Ghana; ^7^ School of Public Health Kwame Nkrumah University of Science and Technology Kumasi Ghana

**Keywords:** abattoir, Ghana, ticks, zoonoses

## Abstract

The high dependence on livestock in Ghana comes with the risk of zoonotic tick‐borne pathogen infection. Abattoir workers are especially at risk due to their frequent contact with livestock that are infested with ticks and tick‐borne pathogens. This study sought to assess the knowledge, attitude and practices of abattoir workers in Kumasi towards ticks and tick‐borne pathogens. A total of 130 workers were recruited from the Kumasi abattoir (92), Suame abattoir (24) and Akwatia Line slaughter slab (14). The respondents were males, mostly aged between 46 and 55 years (36.2%), had no form of education (43.8%) and had >10 years (68.5%) of working experience. Given that a significant number of the workers handled live animals (95.4%) or dead animals or animal parts (87.7%), were involved in slaughtering (69.2%) and had experienced tick bites (81.5%), there was a risk of tick‐borne pathogen infection. However, only 35.4% thought humans can get diseases from tick bites, and 58.5% of the respondents believed tick bites lead to the development of a tick‐borne disease. It was observed that education (*p* = 0.008) had a significant influence on the risk of zoonotic tick‐borne pathogen infection, with 52% of the respondents with no education believing humans cannot get infections from ticks. The findings of this study indicate that the abattoir workers are at risk of zoonotic tick‐borne infections, and there is a need for frequent education as well as the adoption of effective tick control and preventive measures.

## Graphical abstract

Education significantly influenced risk perception (*p* = 0.008); overall, 52% of respondents without formal education denied the possibility of human tick‐borne infections, whereas 44% with basic education recognized this risk. Most respondents (81.5%) experienced tick bites. Additionally, 54.6% did not believe livestock amplify tick‐borne pathogens or transmit them to humans.

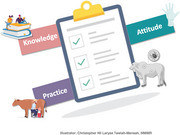

## 1 Introduction

Ticks are a common problem that is thought to pose a serious risk to livestock and human health, which has a substantial financial impact. In the future, zoonotic tick‐borne diseases are predicted to pose a major concern to public health due to their expanded geographic spread and infection rates [1]. Humans can get diseases transmitted by ticks by being bitten by an infected tick or by touching an infected animal [2]. Like in many underdeveloped nations, Ghana's reliance on livestock for sustenance and income puts some groups, especially those who work in abattoirs, at higher risk of contracting zoonoses spread by ticks. These employees frequently work with tick‐infested animals, exposing them to infectious zoonotic pathogens such as *Rickettsia* species, *Coxiella burnetii*, and the Crimean‐Congo haemorrhagic fever virus [3, 4, 5]. In the livestock supply chain, abattoirs are crucial locations where employees are regularly exposed to animal tissues, blood and potentially harmful ectoparasites [6]. In these environments, the danger of zoonotic disease transmission is further increased by inadequate veterinary supervision, poor hygiene and limited use of personal protective equipment. Because pre‐ and post‐slaughter inspections may not be rigorous, there is worry that unregulated abattoirs have a higher incidence of occupational health issues, including zoonoses [7]. Effective prevention and control methods can also be undermined by workers’ lack of knowledge and comprehension of tick‐borne diseases, which can prolong the infection cycle.

Studies in Ghana have reported the circulation of tick‐borne pathogens of zoonotic and veterinary importance [8, 9, 10, 11, 12]. Because livestock are often slaughtered in large numbers at the abattoirs, there is a risk of zoonotic infections. It is important to note that previous studies in the Kumasi abattoir have reported the exposure and occurrence of zoonotic tick‐borne pathogens in abattoir workers, ticks and slaughtered livestock [13, 14, 15]. Despite the acknowledged occupational threat, little is known about Ghanaian abattoir workers’ knowledge, attitudes and behaviours regarding ticks and tick‐borne diseases. The purpose of this study was to close this knowledge gap by evaluating the behavioural practices, attitudes and awareness of Kumasi abattoir workers regarding ticks and tick‐borne diseases. The results will guide focused intervention initiatives to lower disease transmission and safeguard occupational health in this susceptible group by identifying risk factors and educational needs. In Ghana, these initiatives are essential for maintaining sustainable agricultural methods, improving animal productivity and protecting public health.

## 2 Methods

This cross‐sectional study was conducted in two abattoirs and a slaughter slab within the Kumasi Municipality of Ghana. The sites were the Kumasi abattoir at Kaase, the Suame abattoir and the Akwatia Line Slaughter Slab at Amakom. At each site, the study was explained to the workers, and their verbal consent was obtained. A structured questionnaire was administered to each worker to assess their knowledge, attitude and practices towards ticks and tick‐borne pathogens. A simple random sampling approach was used when administering the questionnaire. Using Epi Info v5, a minimum of 50 abattoir workers were required to complete the questionnaires. The sample size was obtained on the basis of the following assumption: a population size of 600 workers, a prevalence rate of 3.7% [16] and a 95% confidence level with a 5% error margin. After giving each participant thorough information about the study's goals, methods, possible risks and benefits, we were able to acquire informed consent. The data were anonymized, stored securely with only the research team having access, and all relevant data protection laws were followed to guarantee participant confidentiality. One hundred and thirty abattoir workers completed the questionnaires. Ethical approval was obtained from the Committee on Human Research Publication and Ethics at the Kwame Nkrumah University of Science and Technology (CHRPE/AP/376/23).

## 3 Statistical Analysis

The data were analysed using R statistical software version 4.5.1. Descriptive statistics were used to summarize demographic characteristics of respondents. Categorical variables were presented as frequencies and percentages. Knowledge was assessed using three key indicators: (1) ability to identify a tick, (2) knowledge that ticks can cause disease in livestock, and (3) knowledge that humans can acquire diseases from tick bites. Associations between demographic variables (abattoir, age, educational level and years of working in the abattoir) and knowledge outcomes were examined using contingency tables with row percentages. Pearson's chi‐square test was applied to assess statistical significance. Where expected cell counts were less than 5, Fisher's exact test was used instead. A *p* value <0.05 was considered statistically significant.

Knowledge questions were scored as 1 for a correct response and 0 for an incorrect response. Each respondent's total score was calculated by summing across the items. The overall knowledge score was then categorized into three levels using Bloom's cut‐off point: 80%–100% = good knowledge, 60%–79% = moderate knowledge, and less than 60% = poor knowledge.

Attitude items were scored on the basis of agreement with positive statements. A correct or favourable response was given a score of 1, and an unfavourable response a score of 0. The total attitude score was summed for each participant. Respondents who scored 50% or more were classified as having a positive attitude, whereas those scoring below 50% were classified as having a negative attitude.

A response reflecting good preventive behaviour was scored as 1, and a poor practice as 0. The total practice score was summed for each participant. Respondents scoring 50% or more were classified as having good practices, whereas those scoring below 50% were classified as having poor practices.

## 4 Results

### 4.1 Demographics

From the questionnaires administered to the abattoir workers, a total of 130 responses were recorded across all three sampling sites: Kumasi abattoir (*n* = 92, 70.7%), Suame abattoir (*n* = 24, 18.5%) and Akwatia Line (*n* = 14, 10.8%) (Table 1). The abattoir workers were males; most of them were aged from 46 to 55 years (36.2%), had no form of education (43.8%) and had over 10 years of working experience in the abattoir (68.5%). The majority of the workers reported coming into contact with live animals (95.4%) and dead animals or animal parts (87.7%) and were involved in the slaughtering of animals (69.2%) (Table S1). Moreover, most of the abattoir workers (97.7%) could identify a tick and reported that the ticks were found on the animals (94.6%). They also reported that cattle are mostly infested with ticks (80%), particularly in the wet season (74.6%), and that the livestock get ticks from fodder grasses (72.3%). Most of the respondents had experienced tick bites (81.5%), suggesting an increased risk of infections.

**TABLE 1:** Demographic characteristics of respondents.

Variables	Abattoir			
	Akwatia Line *N* = 14	Suame *N* = 24	Kumasi *N *= 92	Total *N* = 130
**Gender**				
Male	14 (10.8)	24 (18.5)	92 (70.7)	130 (100.0)
**Age (in years)**				
18–25	0 (0.0)	1 (100.0)	0 (0.0)	1 (0.8)
26–35	2 (8.0)	6 (24.0)	17 (68.0)	25 (19.2)
36–45	5 (12.5)	7 (17.5)	28 (70.0)	40 (30.8)
46–55	4 (8.5)	8 (17.0)	35 (74.5)	47 (36.2)
>56	3 (17.6)	2 (11.8)	12 (70.6)	17 (13.1)
**Educational level**				
None	5 (8.8)	13 (22.8)	39 (68.4)	57 (43.8)
Basic	4 (8.2)	8 (16.3)	37 (75.5)	49 (37.7)
Secondary	3 (18.8)	2 (12.5)	11 (68.8)	16 (12.3)
Tertiary	2 (25.0)	1 (12.5)	5 (62.5)	8 (6.2)
**Years working**				
<1	0 (0.0)	0 (0.0)	3 (100.0)	3 (2.3)
1–5	1 (5.6)	5 (27.8)	12 (66.7)	18 (13.8)
6–10	2 (10.0)	6 (30.0)	12 (60.0)	20 (15.4)
>10	11 (12.4)	13 (14.6)	65 (73.0)	89 (68.5)

### 4.2 Association Between Demographics, Knowledge, Attitude and Practices

It was observed that education level was significantly associated with the knowledge of the abattoir workers (*p* = 0.036), with 64.9% of those with no education having moderate knowledge on ticks and tick‐borne diseases (Table 2). No significant association was observed between the demographic characteristics and the attitudes of the abattoir workers (Table 3). However, it was observed that those with some levels of education were more likely to engage in good practices within the abattoir (*p* = 0.0304) (Table 4).

**TABLE 2:** Demographic characteristics and knowledge.

Demographic variables	Poor *n* (%)	Moderate *n* (%)	Good *n* (%)	*p* value
**Abattoir**				**0.090**
Akwatia Line	2 (14.3)	7 (50.0)	5 (35.7)	
Suame	2 (8.3)	16 (66.7)	6 (25.0)	
Kumasi	29 (31.5)	46 (50.0)	17 (18.5)	
**Age (in years)**				**0.595**
18–25	0 (0.0)	1 (100.0)	0 (0.0)	
26–35	7 (28.0)	14 (56.0)	4 (16.0)	
36–45	12 (30.0)	16 (40.0)	12 (30.0)	
46–55	9 (19.1)	28 (59.6)	10 (21.3)	
>56	5 (29.4)	10 (58.8)	2 (11.8)	
**Educational level**				0.036*
None	13 (22.8)	37 (64.9)	7 (12.3)	
Basic	13 (26.5)	21 (42.9)	15 (30.6)	
Secondary	7 (43.8)	7 (43.8)	2 (12.5)	
Tertiary	0 (0.0)	4 (50.0)	4 (50.0)	
**Years working**				**0.165**
<1	0 (0.0)	2 (66.7)	1 (33.3)	
1–5	3 (16.7)	14 (77.8)	1 (5.6)	
6–10	7 (35.0)	7 (35.0)	6 (30.0)	
>10	23 (25.8)	46 (51.7)	20 (22.5)	

* statistically significant.

**TABLE 3:** Demographic characteristics and attitudes.

Demographic variables	Negative *n* (%)	Positive *n* (%)	*p* value
**Abattoir**			0.0648
Akwatia Line	2 (14.3)	12 (85.7)	
Suame	9 (37.5)	15 (62.5)	
Kumasi	15 (16.3)	77 (83.7)	
**Age (in years)**	0 (0.0)	1 (100.0)	0.5951
18–25	4 (16.0)	21 (84.0)	
26–35	11 (27.5)	29 (72.5)	
36–45	7 (14.9)	40 (85.1)	
46–55	4 (23.5)	13 (76.5)	
>56	0 (0.0)	1 (100.0)	
**Educational level**			0.9278
None	12 (21.1)	45 (78.9)	
Basic	9 (18.4)	40 (81.6)	
Secondary	4 (25.0)	12 (75.0)	
Tertiary	1 (12.5)	7 (87.5)	
**Years working**			0.702
<1	1 (33.3)	2 (66.7)	
1–5	3 (16.7)	15 (83.3)	
6–10	5 (25.0)	15 (75.0)	
>10	17 (19.1)	72 (80.9)	

**TABLE 4:** Demographic characteristics and practices.

Demographic variables	Poor *n* (%)	Good *n* (%)	*p* value
**Abattoir**			**0.1305**
Akwatia Line	7 (50.0)	7 (50.0)	
Suame	17 (70.8)	7 (29.2)	
Kumasi	44 (47.8)	48 (52.2)	
**Age (in years)**			**0.1499**
18–25	0 (0.0)	1 (100.0)	
26–35	11 (44.0)	14 (56.0)	
36–45	26 (65.0)	14 (35.0)	
46–55	25 (53.2)	22 (46.8)	
>56	6 (35.3)	11 (64.7)	
**Educational level**			0.0304*
None	36 (63.2)	21 (36.8)	
Basic	20 (40.8)	29 (59.2)	
Secondary	10 (62.5)	6 (37.5)	
Tertiary	2 (25.0)	6 (75.0)	
**Years working**			**0.1845**
<1	0 (0.0)	3 (100.0)	
1–5	8 (44.4)	10 (55.6)	
6–10	13 (65.0)	7 (35.0)	
>10	47 (52.8)	42 (47.2)	

* statistically significant.

### 4.3 Association Between Examined Variables and Perception of Human Infections From Ticks

Although only 58.5% of the respondents believed tick bites lead to the development of a tick‐borne disease, only 35.4% thought humans can get diseases from the tick bites. A comparison was made between the variables examined and the workers’ thoughts on whether humans get diseases from ticks or not. Age (*p* = 0.49) and years of work experience (*p* = 0.34) did not seem to correlate with thoughts about getting tick‐borne diseases. Furthermore, the slaughtering of animals (*p* = 0.74) and coming into contact with live (*p* = 0.67) or dead animals/animal parts (*p* = 0.45) did not have any influence on the belief that humans can or cannot get diseases from ticks. However, a significant association was seen between education and workers who responded yes or no to humans acquiring diseases from ticks (*p* = 0.008). Even though workers who believed humans could get diseases from ticks had basic education (44%), more of those who thought humans could not get diseases from ticks had no education (52%) (Table S2). Again, it was observed that the workers who believed humans could get diseases from ticks were more likely to collect animal blood (*p* = 0.025). Moreover, a significant number of the workers (*p* < 0.001) who believed humans can get diseases from ticks also thought that tick bites can lead to the development of a tick‐borne disease (87%). A significant number of the abattoir workers (55%) reported pain and irritation (*p* = 0.014) from tick bites, although they did not believe humans can get diseases from ticks. It was also observed that those who believed humans can get diseases from ticks had noticed an increase in the level of tick infestation in the abattoir (*p* < 0.001), thought that livestock can get diseases from the ticks (*p* < 0.001), had heard about tick‐borne diseases in livestock (*p *= 0.003) and also thought that livestock can amplify tick‐borne diseases and contribute to its transmission to humans (*p* < 0.001). The abattoir workers who believed humans could get diseases from ticks were worried about tick bites (*p* = 0.002). To prevent tick bites, they employed measures such as the wearing of long pants/trousers (*p* = 0.010) or long‐sleeved shirts (*p* = 0.013), performing tick checks on themselves (*p* = 0.005) and wearing gloves when handling the livestock or livestock products (*p* = 0.034).

## 5 Discussion

Knowing the effects of ticks and tick‐borne diseases on people and animals is crucial since awareness of the risk of infection often encourages improved hygiene habits that reduce the spread of the disease. In this study, the abattoir workers had a fair knowledge of ticks. Most of the workers could identify ticks and further indicated cattle to be mostly infested by these ticks, particularly in the wet season. This supports studies in Ghana that have reported cattle to be mostly infested with ticks compared to other livestock such as goats and sheep [17, 18, 19].

It was observed that the majority of the abattoir workers did not think that humans could get diseases from ticks. Furthermore, age and years of working experience did not seem to correlate with thoughts about getting tick‐borne diseases. This could be because most of the workers had no formal education. Humans get infected with tick‐borne pathogens through tick bites or coming into contact with infected animals or animal products [2]. Although a significant number of the abattoir workers had experienced tick bites and were at risk of infections, a majority did not believe humans could get diseases from ticks. They were not worried about tick bites, although they experienced pain and irritation from the bites. It was thus not surprising when the findings of this study showed that most of the abattoir workers did not engage in activities that reduce tick bites and prevent zoonotic pathogen spread. *Rickettsia* is transmitted to humans through tick bites or excrement from ticks that contaminate wounds from scratching [20]. Studies in the Kumasi abattoir have reported the occurrence of *Rickettsia* pathogens *R. africae* and *R. aeschlimannii* in ticks [11, 15] and humans [13]. Another zoonotic tick‐borne pathogen that can spread through a tick bite is CCHFV [21]. This pathogen has been reported in ticks from the Kumasi abattoir with pathogen exposure in the workers [14]. It is obvious that the abattoir workers are at risk of infections and need to adopt tick bite preventive measures, such as the wearing of protective clothing and conducting regular tick checks [22].

It was also observed in this study that most of the abattoir workers handled live animals, dead animals or animal parts and were involved in the slaughtering of animals. This puts the workers at risk of infections because some tick‐borne pathogens are transmitted through coming into direct contact with infected animals or animal products such as milk and bodily fluids [23, 24]. However, most of the workers did not think livestock could get diseases from the ticks, did not know about tick‐borne diseases in livestock and thought livestock could not amplify tick‐borne diseases and contribute to their transmission to humans. This can be compared to a study in Kenya where only a few slaughterhouse workers were aware of the role animals play in disease transmission to humans [25]. A lack of knowledge about livestock's role as zoonotic disease reservoirs and amplifiers might impede the implementation of efficient tick control measures and encourage dangerous practices such as unprotected direct contact with infected animals. Abattoir workers must acknowledge that a significant percentage of newly emerging infectious diseases have their origins in animals and that 60% of human infectious diseases are zoonotic [26]. Workplace cleanliness and appropriate meat safety procedures should also be taught to abattoir workers through the creation and implementation of training programmes [6].

The study's findings have significant ramifications for public health in Ghana, especially for the group of abattoir workers who are exposed on the job. Despite extensive exposure to ticks and potentially infected livestock, workers’ significant knowledge gaps and misconceptions about tick‐borne infections underscore the urgent need for improved health education and awareness initiatives at abattoirs. Enhancing knowledge of the threats of zoonotic transmission and the significance of environmental tick control and preventive actions, such as wearing personal protective equipment, should be the main goals of these programmes. Routine training, the use of protective clothing and the implementation of participatory surveillance systems could all be facilitated by enhancing partnerships among public health, veterinary services and abattoir management. To lower spillover risks, protect worker and community health and increase animal productivity, Ghana's livestock industry has to use such integrated One Health initiatives.

It is important to take into account some limitations when interpreting the study's findings. The cross‐sectional study design makes it challenging to evaluate causality or changes over time because it only records a snapshot of knowledge, attitudes and practices at one particular moment in time. Potential biases like recall bias and social desirability bias are introduced by the use of self‐reported questionnaire data, which may compromise the honesty of reported experiences and behaviours. Furthermore, conclusions about infection risk are still indirect and inferential because this study did not combine behavioural data with direct pathogen detection in human or animal samples. A more thorough grasp of the dangers to occupational health and the effectiveness of educational interventions might be possible with future research that includes longitudinal designs, serological or molecular screening and larger population samples.

## 6 Conclusion

This study reveals significant gaps in knowledge, perception and practices of Kumasi abattoir workers regarding ticks and tick‐borne pathogens. A zoonotic infection risk is created by the combination of systemic workplace vulnerabilities, regular tick exposure, ignorance and misconceptions about disease transmission. It is essential to address these complex issues through policy change, infrastructure improvement, education and participatory surveillance to protect worker health and lessen the overall public health impact of zoonotic diseases.

## Author Contributions

**Seth Offei Addo**: conceptualization, writing–original draft, investigation. Margaret Addo: investigation, writing–review and editing. **Michael E. DeWitt:** investigation, formal analysis, writing–review and editing. **Christopher Nii Laryea Tawiah‐Mensah:** investigation, writing–review and editing. **Stacy Amoah:** investigation, writing–review and editing. **Patrick Kwasi Obuam:** investigation, formal analysis, writing–review and editing. **Nancy Martekai Unicorn:** investigation, writing–review and editing. **Emmanuella Tiwaa Kyeremateng:** investigation, writing–review and editing. **Genevieve Desewu:** investigation, writing–review and editing. **John Asiedu Larbi:** Supervision, investigation, writing–review and editing.

## Conflicts of Interest

The authors declare no conflicts of interest.

## Supporting information



Table S1 Responses of participants to the various questions.

Table S2 Examined variables and perception of human infections from ticks.

## Data Availability

This article contains all the data supporting the findings.
