# Supplementary material for: Knowledge, Attitude and Practices of Abattoir Workers in Kumasi Towards Ticks and Tick‐Borne Pathogens
Source: Public Health Chall. 2025 Nov 14;4(4):e70167. doi: 10.1002/puh2.70167 (PMC12617350; doi:10.1002/puh2.70167)
Supplement: Supplementary file 1 — Table S1 Responses of participants to the various questions. [file PUH2-4-e70167-s002.docx]

**Table S1: Responses of participants to the various questions**

| **Questions** | **Frequency** | **Percentage (%)** |
| --- | --- | --- |
| Do you come into contact with live animals? |  |  |
| Yes | 124 | 95.4 |
| No | 6 | 4.6 |
| Do you come into contact with dead animals or animal parts? |  |  |
| Yes | 114 | 87.7 |
| No | 16 | 12.3 |
| Slaughter animals? |  |  |
| Yes | 90 | 69.2 |
| No | 40 | 30.8 |
| Collect animal blood? |  |  |
| Yes | 38 | 29.2 |
| No | 92 | 70.8 |
| Can you identify a tick? |  |  |
| Yes | 127 | 97.7 |
| No | 3 | 2.3 |
| Can tick bite lead to the development of a tick-borne disease? |  |  |
| Yes | 76 | 58.5 |
| No | 54 | 41.5 |
| Do you think ticks always stay on the body of a livestock unless removed? |  |  |
| Yes | 42 | 32.3 |
| No | 77 | 59.2 |
| Don’t know | 11 | 8.5 |
| Do you think humans can get diseases from the tick bites? |  |  |
| Yes | 46 | 35.4 |
| No | 27 | 20.8 |
| Don’t know | 57 | 43.8 |
| Do you think the livestock can get diseases from the ticks? |  |  |
| Yes | 57 | 43.8 |
| No | 19 | 14.6 |
| Don’t know | 54 | 41.5 |
| Have you heard of any tick-borne diseases in livestock? |  |  |
| Yes | 20 | 15.4 |
| No | 110 | 84.6 |
| Where do you commonly find ticks? |  |  |
|  | On animals only | 123 (94.6) |
|  | On animals and ground | 3 (2.3) |
|  | On animals and pastureland | 3 (2.3) |
|  | On ground only | 1 (0.8) |
| Which livestock are commonly infested with ticks? |  |  |
|  | Cattle | 104 (80.0) |
|  | Goat | 15 (11.5) |
|  | Sheep | 11 (8.5) |
| Which season do you commonly see ticks? |  |  |
|  | Dry | 3 (2.3) |
|  | Wet | 97 (74.6) |
|  | Both Wet and Dry | 30 (23.1) |
| Where do you think livestock get ticks from? |  |  |
|  | Fodder grasses | 94 (72.3) |
|  | Bedding materials | 8 (6.2) |
|  | Both bedding and fodder | 17 (13.1) |
|  | Don’t know | 11 (8.5) |
| Which age group of livestock are most affected? |  |  |
|  | <1 | 33 (25.4) |
|  | 01-May | 72 (55.4) |
|  | 06-Oct | 44 (33.8) |
|  | >10 | 31 (23.8) |
|  | Don’t know | 7 (5.4) |
| Which body part of livestock is commonly infested? |  |  |
|  | Anus and perianal region | 53 (40.8) |
|  | Dewlap | 9 (6.9) |
|  | Feet | 34 (26.2) |
|  | Head region | 2 (1.5) |
|  | Neck region | 8 (6.2) |
|  | Others (belly and limbs) | 33 (25.4) |
|  | Scrotum and udder | 125 (96.2) |
| Have you been bitten by a tick before? |  |  |
|  | Yes | 106 (81.5) |
|  | No | 24 (18.5) |
|  |  |  |
| What are the clinical signs of tick bite? |  | **n=127** |
|  | Fever and headache | 1 (0.8) |
|  | Pain and irritation | 89 (70.1) |
|  | Rash and swelling | 26 (20.5) |
|  | No sign | 11 (8.7) |
| What are the health and production impacts of tick infestation? |  |  |
|  | Loss of weight | 62 (47.7) |
|  | Hide damage | 43 (33.1) |
|  | Bite wound | 40 (30.8) |
|  | Loss of appetite | 38 (29.2) |
|  | Anaemia | 35 (26.9) |
|  | Loss of production | 2 (1.5) |
|  | Fever | 2 (1.5) |
|  | Don’t know | 9 (6.9) |
| Source of information about tick-borne diseases |  | **n=22** |
|  | Media (including social media) | 1 (4.5) |
|  | Veterinarian | 11 (50.0) |
|  | Friends | 1 (4.5) |
|  | Family members | 1 (4.5) |
|  | Abattoirs’ training program | 8 (36.4) |
| Worried about being bitten by ticks. |  |  |
| Yes | 55 | 42.7 |
| No | 75 | 57.7 |
| Interested in taking measures to prevent tick bites. |  |  |
| Yes | 101 | 77.7 |
| No | 29 | 22.3 |
| Apply insect repellent to prevent/avoid tick bites. |  |  |
| Yes | 121 | 93.1 |
| No | 9 | 6.9 |
| Wear long pants/trousers to avoid tick bites. |  |  |
| Yes | 62 | 47.7 |
| No | 68 | 52.3 |
| Wear long-sleeved shirts to prevent/avoid tick bites. |  |  |
| Yes | 60 | 46.2 |
| No | 70 | 53.8 |
| Perform tick checks on myself. |  |  |
| Yes | 66 | 50.8 |
| No | 64 | 49.2 |
| Shower immediately I get home after coming into contact with the livestock or their body parts. |  |  |
| Yes | 114 | 87.7 |
| No | 16 | 12.3 |
| Wear gloves when handling the livestock or livestock products. |  |  |
| Yes | 14 | 10.8 |
| No | 116 | 89.2 |
