# Supplementary material for: Knowledge, Attitude and Practices of Abattoir Workers in Kumasi Towards Ticks and Tick‐Borne Pathogens
Source: Public Health Chall. 2025 Nov 14;4(4):e70167. doi: 10.1002/puh2.70167 (PMC12617350; doi:10.1002/puh2.70167)
Supplement: Supplementary file 2 — Table S2 Examined variables and perception of human infections from ticks. [file PUH2-4-e70167-s001.docx]

Table S2: **Examined variables and perception of human infections from ticks**

| **Characteristic** | **Overall**, N = 130 | **No, humans cannot get diseases from ticks**, N = 84 | **Yes, humans can get diseases from ticks**, N = 46 | **p-value**^1^ |
| --- | --- | --- | --- | --- |
| **Abattoir name, n (%)** |  |  |  | 0.77 |
| Akwatia Line | 14 (11) | 8 (9.5) | 6 (13) |  |
| Kumasi Abattoir | 92 (71) | 61 (73) | 31 (67) |  |
| Suame Abattoir | 24 (18) | 15 (18) | 9 (20) |  |
| **Age (years), n (%)** |  |  |  | 0.49 |
| 18-25 | 1 (0.8) | 0 (0) | 1 (2.2) |  |
| 26-35 | 25 (19) | 18 (21) | 7 (15) |  |
| 36-45 | 40 (31) | 23 (27) | 17 (37) |  |
| 46-55 | 47 (36) | 32 (38) | 15 (33) |  |
| 56 and above | 17 (13) | 11 (13) | 6 (13) |  |
| **Gender, n (%)** |  |  |  |  |
| Male | 130 (100) | 84 (100) | 46 (100) |  |
| **Educational Level, n (%)** |  |  |  | **0.008** |
| None | 56 (43) | 44 (52) | 12 (27) |  |
| Basic | 49 (38) | 29 (35) | 20 (44) |  |
| Secondary | 16 (12) | 9 (11) | 7 (16) |  |
| Tertiary | 8 (6.2) | 2 (2.4) | 6 (13) |  |
| Unknown | 1 | 0 | 1 |  |
| **Number of years working in the Abattoir, n (%)** |  |  |  | 0.34 |
| <1 | 3 (2.3) | 1 (1.2) | 2 (4.3) |  |
| 1-5 | 18 (14) | 14 (17) | 4 (8.7) |  |
| 6-10 | 20 (15) | 14 (17) | 6 (13) |  |
| >10 | 89 (68) | 55 (65) | 34 (74) |  |
| **Do you come into contact with live animals?, n (%)** | 124 (95) | 81 (96) | 43 (93) | 0.67 |
| **Do you come into contact with dead animals or animal parts?, n (%)** | 114 (88) | 75 (89) | 39 (85) | 0.45 |
| **Slaughter animals?, n (%)** | 90 (69) | 59 (70) | 31 (67) | 0.74 |
| **Collect animal blood?, n (%)** | 38 (29) | 19 (23) | 19 (41) | **0.025** |
| **Can you identify a tick?, n (%)** | 127 (98) | 82 (98) | 45 (98) | >0.99 |
| **Where do you commonly find ticks?, n (%)** |  |  |  | 0.29 |
| On the animals | 123 (95) | 81 (96) | 42 (91) |  |
| On the animals, In the pastureland | 3 (2.3) | 1 (1.2) | 2 (4.3) |  |
| On the animals, On the ground | 3 (2.3) | 2 (2.4) | 1 (2.2) |  |
| On the ground | 1 (0.8) | 0 (0) | 1 (2.2) |  |
| **Which livestock are commonly infested with ticks?, n (%)** |  |  |  | 0.43 |
| Cattle | 113 (87) | 74 (88) | 39 (85) |  |
| Cattle, Goat | 6 (4.6) | 5 (6.0) | 1 (2.2) |  |
| Cattle, Sheep | 2 (1.5) | 1 (1.2) | 1 (2.2) |  |
| Cattle, Sheep, Goat | 9 (6.9) | 4 (4.8) | 5 (11) |  |
| **Which season do you commonly see the ticks on the livestock?, n (%)** |  |  |  | 0.59 |
| Dry | 3 (2.3) | 1 (1.2) | 2 (4.3) |  |
| Wet | 97 (75) | 63 (75) | 34 (74) |  |
| Wet and Dry | 30 (23) | 20 (24) | 10 (22) |  |
| **Where do you think the livestock get ticks from?, n (%)** |  |  |  | 0.062 |
| Don’t know | 11 (8.5) | 5 (6.0) | 6 (13) |  |
| From fodder grasses | 94 (72) | 67 (80) | 27 (59) |  |
| From the bedding materials | 8 (6.2) | 3 (3.6) | 5 (11) |  |
| From the bedding materials, From fodder grasses | 17 (13) | 9 (11) | 8 (17) |  |
| **Can Tick bite lead to the development of a tick-borne disease?, n (%)** | 76 (58) | 36 (43) | 40 (87) | **<0.001** |
| **Which age group of the livestock are most affected by tick infestation?, n (%)** |  |  |  |  |
| <1 | 20 (15) | 9 (11) | 11 (24) |  |
| <1, >10 | 2 (1.5) | 2 (2.4) | 0 (0) |  |
| <1, 1-5 | 5 (3.8) | 1 (1.2) | 4 (8.7) |  |
| <1, 1-5, 6-10, >10 | 6 (4.6) | 2 (2.4) | 4 (8.7) |  |
| >10 | 2 (1.5) | 1 (1.2) | 1 (2.2) |  |
| 1-5 | 47 (36) | 32 (38) | 15 (33) |  |
| 1-5, >10 | 3 (2.3) | 2 (2.4) | 1 (2.2) |  |
| 1-5, 6-10, >10 | 11 (8.5) | 9 (11) | 2 (4.3) |  |
| 6-10 | 20 (15) | 18 (21) | 2 (4.3) |  |
| 6-10, >10 | 7 (5.4) | 4 (4.8) | 3 (6.5) |  |
| Don’t know | 7 (5.4) | 4 (4.8) | 3 (6.5) |  |
| **Which part of the body on the livestock do you commonly find ticks?, n (%)** |  |  |  | **0.037** |
| Anus and perianal region | 1 (0.8) | 1 (1.2) | 0 (0) |  |
| Head region | 1 (0.8) | 0 (0) | 1 (2.2) |  |
| Head region, Scrotum and udder, Anus and perianal region | 1 (0.8) | 0 (0) | 1 (2.2) |  |
| Neck region | 1 (0.8) | 0 (0) | 1 (2.2) |  |
| Neck region, Anus and perianal region | 1 (0.8) | 0 (0) | 1 (2.2) |  |
| Neck region, Scrotum and udder, Anus and perianal region, Dewlap, Others (belly and limbs) | 1 (0.8) | 1 (1.2) | 0 (0) |  |
| Neck region, Scrotum and udder, Anus and perianal region, Others (belly and limbs) | 2 (1.5) | 1 (1.2) | 1 (2.2) |  |
| Neck region, Scrotum and udder, Dewlap | 1 (0.8) | 1 (1.2) | 0 (0) |  |
| Neck region, Scrotum and udder, Feet (i.e., between or just above the hooves) | 1 (0.8) | 0 (0) | 1 (2.2) |  |
| Neck region, Scrotum and udder, Others (belly and limbs) | 1 (0.8) | 1 (1.2) | 0 (0) |  |
| Others (belly and limbs) | 1 (0.8) | 1 (1.2) | 0 (0) |  |
| Scrotum and udder | 51 (39) | 33 (39) | 18 (39) |  |
| Scrotum and udder, Anus and perianal region | 10 (7.7) | 5 (6.0) | 5 (11) |  |
| Scrotum and udder, Anus and perianal region, Dewlap, Others (belly and limbs) | 7 (5.4) | 6 (7.1) | 1 (2.2) |  |
| Scrotum and udder, Anus and perianal region, Feet (i.e., between or just above the hooves) | 11 (8.5) | 5 (6.0) | 6 (13) |  |
| Scrotum and udder, Anus and perianal region, Feet (i.e., between or just above the hooves), Others (belly and limbs) | 4 (3.1) | 2 (2.4) | 2 (4.3) |  |
| Scrotum and udder, Anus and perianal region, Others (belly and limbs) | 15 (12) | 9 (11) | 6 (13) |  |
| Scrotum and udder, Feet (i.e., between or just above the hooves) | 18 (14) | 17 (20) | 1 (2.2) |  |
| Scrotum and udder, Others (belly and limbs) | 2 (1.5) | 1 (1.2) | 1 (2.2) |  |
| **Do you think ticks always stay on the body of a livestock unless removed?, n (%)** |  |  |  | 0.13 |
| Don’t know | 11 (8.5) | 10 (12) | 1 (2.2) |  |
| No | 77 (59) | 50 (60) | 27 (59) |  |
| Yes | 41 (32) | 23 (27) | 18 (39) |  |
| Yes, No | 1 (0.8) | 1 (1.2) | 0 (0) |  |
| **Have you been bitten by a tick before?, n (%)** | 106 (82) | 72 (86) | 34 (74) | 0.10 |
| **What were the clinical signs of the tick bite?, n (%)** |  |  |  | **0.014** |
| No symptoms | 11 (8.5) | 11 (13) | 0 (0) |  |
| Not applicable | 26 (20) | 15 (18) | 11 (24) |  |
| Pain and irritation | 67 (52) | 46 (55) | 21 (46) |  |
| Pain and irritation, Rash and swelling around the bite | 21 (16) | 10 (12) | 11 (24) |  |
| Pain and irritation, Rash and swelling around the bite, Fever and headache | 1 (0.8) | 0 (0) | 1 (2.2) |  |
| Rash and swelling around the bite | 4 (3.1) | 2 (2.4) | 2 (4.3) |  |
| **Have you noticed an increase in the level of tick infestation in the abattoir?, n (%)** | 60 (46) | 28 (33) | 32 (70) | **<0.001** |
| **What do you think is the health and production impacts of tick infestation in livestock?, n (%)** |  |  |  |  |
| Anaemia | 7 (5.4) | 1 (1.2) | 6 (13) |  |
| Anaemia, Bite wound | 14 (11) | 7 (8.3) | 7 (15) |  |
| Anaemia, Bite wound, Loss of appetite, Loss of weight, Loss of production | 1 (0.8) | 1 (1.2) | 0 (0) |  |
| Anaemia, Bite wound, Loss of weight | 1 (0.8) | 1 (1.2) | 0 (0) |  |
| Anaemia, Hide damage | 1 (0.8) | 0 (0) | 1 (2.2) |  |
| Anaemia, Loss of appetite | 2 (1.5) | 0 (0) | 2 (4.3) |  |
| Anaemia, Loss of appetite, Loss of weight | 5 (3.8) | 4 (4.8) | 1 (2.2) |  |
| Anaemia, Loss of appetite, Loss of weight, Fever | 1 (0.8) | 1 (1.2) | 0 (0) |  |
| Anaemia, Loss of weight | 2 (1.5) | 2 (2.4) | 0 (0) |  |
| Anaemia, Loss of weight, Hide damage | 1 (0.8) | 0 (0) | 1 (2.2) |  |
| Bite wound | 11 (8.5) | 7 (8.3) | 4 (8.7) |  |
| Bite wound, Hide damage | 8 (6.2) | 5 (6.0) | 3 (6.5) |  |
| Bite wound, Loss of appetite, Fever, Hide damage, Loss of production | 1 (0.8) | 0 (0) | 1 (2.2) |  |
| Bite wound, Loss of appetite, Loss of weight | 1 (0.8) | 1 (1.2) | 0 (0) |  |
| Bite wound, Loss of weight | 1 (0.8) | 1 (1.2) | 0 (0) |  |
| Bite wound, Loss of weight, Hide damage | 2 (1.5) | 2 (2.4) | 0 (0) |  |
| Don’t know | 8 (6.2) | 5 (6.0) | 3 (6.5) |  |
| Hide damage | 13 (10) | 7 (8.3) | 6 (13) |  |
| Loss of appetite | 1 (0.8) | 0 (0) | 1 (2.2) |  |
| Loss of appetite, Hide damage | 2 (1.5) | 1 (1.2) | 1 (2.2) |  |
| Loss of appetite, Loss of weight | 18 (14) | 15 (18) | 3 (6.5) |  |
| Loss of appetite, Loss of weight, Hide damage | 6 (4.6) | 3 (3.6) | 3 (6.5) |  |
| Loss of weight | 13 (10) | 12 (14) | 1 (2.2) |  |
| Loss of weight, Don’t know | 1 (0.8) | 1 (1.2) | 0 (0) |  |
| Loss of weight, Hide damage | 9 (6.9) | 7 (8.3) | 2 (4.3) |  |
| **Do you think the livestock can get diseases from the ticks?, n (%)** |  |  |  | **<0.001** |
| Don’t know | 54 (42) | 48 (57) | 6 (13) |  |
| No | 19 (15) | 17 (20) | 2 (4.3) |  |
| Yes | 57 (44) | 19 (23) | 38 (83) |  |
| **Have you heard of any tick-borne diseases in livestock?, n (%)** | 20 (15) | 7 (8.3) | 13 (28) | **0.003** |
| **If yes, from where did you hear about the tick-borne diseases in livestock?, n (%)** |  |  |  | 0.10 |
| Abattoirs’ training program | 8 (6.2) | 3 (3.6) | 5 (11) |  |
| Family members | 1 (0.8) | 1 (1.2) | 0 (0) |  |
| Friends | 1 (0.8) | 1 (1.2) | 0 (0) |  |
| Media (including social media) | 1 (0.8) | 0 (0) | 1 (2.2) |  |
| Not applicable | 108 (83) | 74 (88) | 34 (74) |  |
| Veterinarian | 11 (8.5) | 5 (6.0) | 6 (13) |  |
| **Do you think livestock can amplify tick-borne diseases and contribute to its transmission to humans?, n (%)** | 59 (45) | 25 (30) | 34 (74) | **<0.001** |
| **Worried about being bitten by ticks., n (%)** | 55 (42) | 27 (32) | 28 (61) | **0.002** |
| **Interested in taking measures to prevent tick bites., n (%)** | 101 (78) | 66 (79) | 35 (76) | 0.74 |
| **I apply insect repellent to prevent/avoid tick bites., n (%)** | 9 (6.9) | 6 (7.1) | 3 (6.5) | >0.99 |
| **I wear long pants/trousers to avoid tick bites., n (%)** | 62 (48) | 33 (39) | 29 (63) | **0.010** |
| **I wear long-sleeved shirts to prevent/avoid tick bites., n (%)** | 60 (46) | 32 (38) | 28 (61) | **0.013** |
| **I perform tick checks on myself., n (%)** | 66 (51) | 35 (42) | 31 (67) | **0.005** |
| **I shower immediately I get home after coming into contact with the livestock or their body parts., n (%)** | 114 (88) | 73 (87) | 41 (89) | 0.71 |
| **I wear gloves when handling livestock or livestock products., n (%)** | 14 (11) | 5 (6.0) | 9 (20) | **0.034** |
| ^1^Fisher's exact test; Pearson's Chi-squared test | | | | |
